# Predicting Recurrent Venous Thromboembolism in Patients With Deep-Vein Thrombosis: Development and Internal Validation of a Potential New Prediction Model (Continu-8)

**DOI:** 10.3389/fcvm.2021.655226

**Published:** 2021-04-06

**Authors:** Michael Nagler, Sander M. J. Van Kuijk, Hugo Ten Cate, Martin H. Prins, Arina J. Ten Cate-Hoek

**Affiliations:** ^1^University Institute of Clinical Chemistry, Inselspital, Bern University Hospital, University of Bern, Bern, Switzerland; ^2^Department of Clinical Epidemiology and Medical Technology Assessment, Maastricht University Medical Centre, Maastricht, Netherlands; ^3^Laboratory of Clinical Thrombosis and Haemostasis, Thrombosis Expertise Center, Cardiovascular Research Institute, Maastricht University Medical Center, Maastricht, Netherlands

**Keywords:** venous thrombosis/epidemiology, venous thrombosis/therapy, venous thrombosis/mortality, risk factors, clinical decision making, health services research

## Abstract

**Background:** Previous prediction models for recurrent thromboembolism (VTE) are often complicated to apply and have not been implemented widely.

**Aim:** To develop and internally validate a potential new prediction model for recurrent VTE that can be used without stopping anticoagulant treatment for D-dimer measurements in patients with provoked and unprovoked DVT.

**Methods:** Cohort data of 479 patients treated in a clinical care pathway at Maastricht University Medical Center were used. Predictors for the Cox proportional hazards model (unprovoked DVT, male gender, factor VIII levels) were derived from literature and using forward selection procedure. The scoring rule was internally validated using bootstrapping techniques and the predictive ability was compared to existing prediction models.

**Results:** Patients were followed for a median of 3.12 years after stopping anticoagulation treatment (IQR 0.78, 3.90). Sixty-four of 479 patients developed recurrent VTE (13%). The scoring rule consisted of unprovoked DVT (yes: 2 points), male sex (yes: 1 point), and factor VIII > 213 % (yes: 2 points) and was categorized into three groups [i.e., low risk (score 0), medium risk (scores 1, 2, or 3) and high risk (scores 4 and 5)]. The concordance statistic was 0.68 (95% CI: 0.61, 0.75).

**Conclusion:** The discriminative ability of the new Continu-8 score was adequate. Future studies shall verify this score in an independent setting without stopping anticoagulation treatment.

## Introduction

Secondary prevention of venous thromboembolism (VTE) is important to improve care in patients with deep vein thrombosis (DVT) or pulmonary embolism (PE) (1–4). VTE is the third most common cardiovascular disease, and it contributes relevantly to the global disease burden (1–3, 5, 6). The estimated incidence is between 1 and 2 per 1,000 person-years and it is associated with a significant morbidity and mortality, both in short-term and long-term perspectives (5, 7, 8). In addition, VTE is associated with substantial healthcare costs (9–11). Though anticoagulation treatment is very effective in treatment and prevention of VTE, it is associated with a significant risk of bleeding complications (12–14); the corresponding case-fatality rate is estimated to be 6% (7). To guide treatment decisions in secondary prevention of VTE, it is important to discriminate those 25% of patients who will recur within 5 years from the 75% of patients who will not (2).

A number of predictors for VTE recurrence have been identified, the presence of reversible risk factors and active cancer being the most relevant (14–16). Consistently, all large cohort studies found an association between the absence of reversible risk factors such as recent surgery, pregnancy and estrogen treatment, and recurrent VTE (2, 14, 17). Men bear a 2-fold risk of VTE recurrence compared to women (18). In addition, elevated D-dimers 1 months after stopping anticoagulation are associated with a high risk of VTE recurrence (19–21). The drawback of a management according to D-dimer levels is that anticoagulation must be stopped at least for 1 month. Factor VIII was studied as another surrogate for an increased coagulation activity by several authors (22–24). More predictors have been suggested in patients without cancer, the applicability in clinical practice is however limited (2). How to combine the predictors optimally is still elusive.

Several prediction models for recurrence of VTE have been developed, yet none of them is strongly recommended so far (14, 25, 26). Rodger and colleagues followed 600 patients with a first, unprovoked proximal VTE for a median of 18 months after stopping anticoagulant treatment and studied 69 predictors using a logistic regression model in males and females (“HERDOO2”) (27, 28). D-dimer, age, body mass index, and post-thrombotic signs were included in the model. However, the risk of overfitting was high (2.5 events per predictor) and the application is limited to women with an unprovoked VTE (25). The prediction rule was validated in a prospective cohort management study (28). Eichinger and colleagues included 929 patients with a first, unprovoked proximal or distal VTE, and followed the patients for a median of 43.3 months after stopping anticoagulation treatment (29). Eight prespecified predictors were studied in 176 recurrent events (22 events per predictor) in a Cox proportional hazards model (the “Vienna prediction model”). Finally, quantitative D-dimer measurements, sex and site of index event were included in the model. The Vienna prediction model was updated (30) and externally validated in two other cohorts with varying results (31, 32). The “DASH” score was derived using individual patient-data from seven prospective studies including patients with a first episode of proximal VTE (33). Six variables obtained from univariate analysis and theoretical considerations were included in a Cox regression analysis and the model was derived from backward selection (40 events per predictor). The final model comprised D-dimer, age, sex, and hormone therapy. The DASH score was externally validated in a retrospective cohort (34).

Several important limitations appear with regard to the existing prediction rules: (1) anticoagulation treatment shall be stopped for measuring D-dimers, (2) the application is difficult, and (3) the application is limited to patients with unprovoked VTE in case of the DASH and HERDOO2 score (25).

With the present investigation, we aimed to develop and internally validate a potential new prediction model for recurrent VTE that can be used in patients with provoked and unprovoked proximal DVT without stopping anticoagulant treatment.

## Methods

### Study Design, Setting and Population

**Figure 1:**
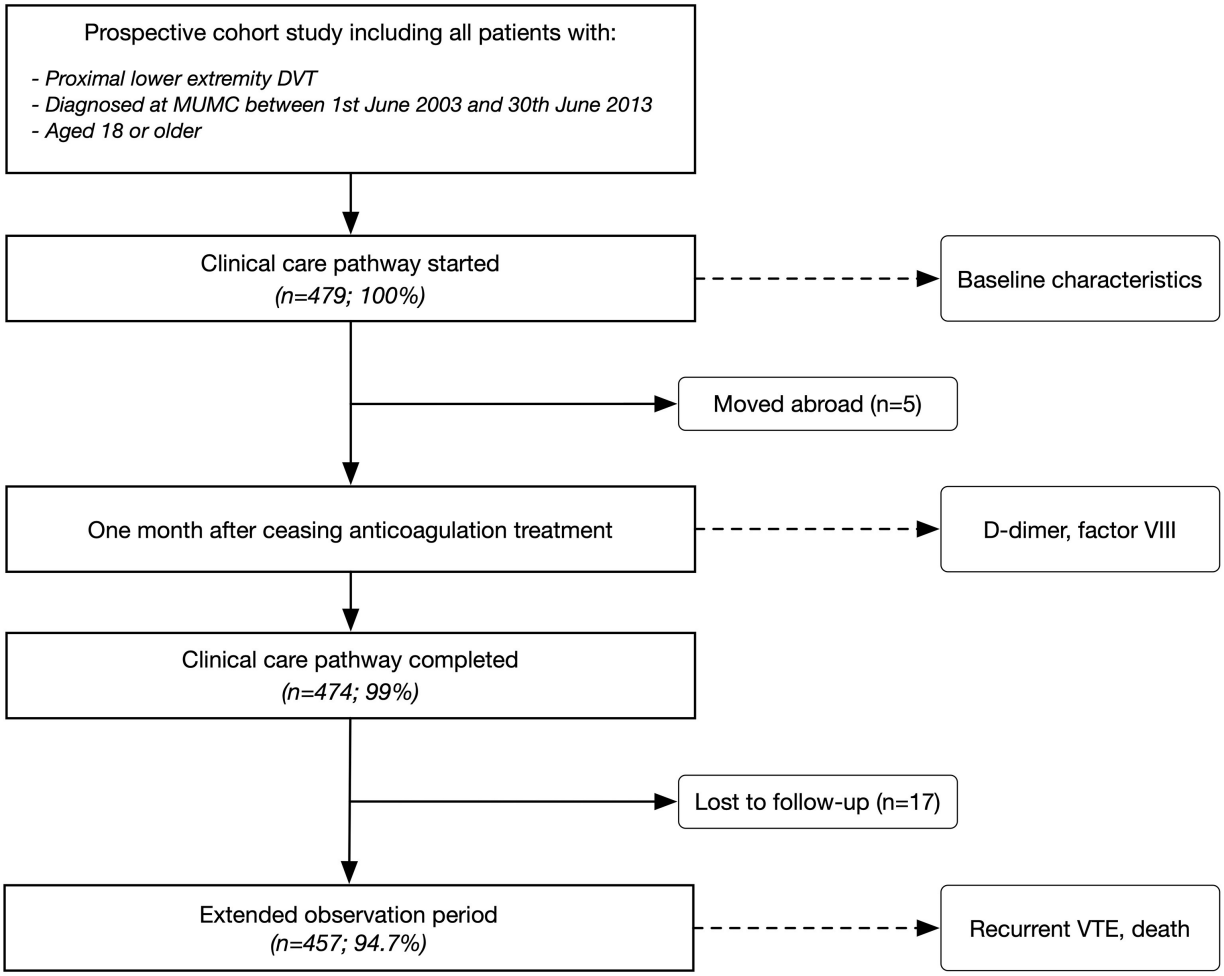
Flow of the patients. MUMC, Maastricht University Medical Center.

Data of a prospective cohort study observing patients with a proximal DVT were used, the details of which have been published (15). All consecutive patients treated within a clinical care pathway (CCP) at Maastricht University Medical Center (MUMC) were included. Inclusion criteria were (a) objectively confirmed first proximal lower extremity DVT (popliteal vein, femoral vein, common femoral vein, iliac vein), (b) diagnosed at MUMC between 1st of June 2003 and 30th of June 2013, and (c) aged 18 or older. Patients were managed in a specialized outpatient unit for 2 years as part of the CCP (Figure 1). Details of the CCP including risk assessment and treatment decisions are described elsewhere (15). Some patient groups were usually not treated within the CCP: distal DVT, pulmonary embolism, and patients with cancer. MUMC is the only tertiary hospital in the province of Limburg, the Netherlands. The study protocol was approved by the appropriate ethical committee (METC 15-4-256) and it was carried out in accordance with the Declaration of Helsinki.

### Collection of Data and Determination of Laboratory Tests

All data were collected prospectively in-line with clinical routine and were recorded in a structured database. Regular visits were scheduled until 24 months. At the first visit of the CCP, structured history was taken as well as physical examination. Clinical risk factors were assessed. Laboratory tests were performed 1 months after stop anticoagulant treatment (e.g., D-dimer, factor VIII). Clinical outcomes were assessed until the last visit. Patients were additionally followed over the course of further outpatient visits and accessing MUMC and general practitioner records. Observer were not aware about outcomes while assessing predictors and predictors while assessing outcomes, respectively. Laboratory tests were conducted at pre-specified time points as previously described (35). A protocol was implemented to ensure adequate pre-analytical conditions (15). D-dimer levels were determined using the Vidas assay (bioMérieux Clinical Diagnostics, Marcy-l'Etoile, France) or Innovance, respectively (Siemens Healthcare, Marburg, Germany). Factor VIII was analyzed using a one-stage assay (Actin FS, Siemens Healthcare, Marburg, Germany) on a Sysmex CA7000 (distributed by Siemens Healthcare, Marburg, Germany).

### Definition of Outcomes and Predictor Variables

We defined recurrent VTE as symptomatic, objectively confirmed proximal or distal DVT, PE, or other venous thrombosis, whereas confirmation was done using compression ultrasound, spiral computed tomography, or ventilation-perfusion lung scanning. A diagnostic work-up was conducted in case of signs and symptoms suggesting VTE as done in routine clinical practice. Recurrence of DVT was defined as (a) a new non-compressible vein in the contralateral leg, (b) a new non-compressible vein of the same leg as the first event (previously unaffected), (c) a clear proximal extension of the known thrombus, or (d) a new non-compressible site of a vein that was effected but previously re-canalized (15, 36–38). D-dimer were defined as positive if above or equal 500 ng/ml. Factor VIII was considered positive if above or equal 213% (80th percentile of the study population). “Unprovoked DVT” was defined as DVT without the presence of a reversible risk factor (20). All other variables investigated in the cohort study were defined previously (20).

### Selection of Predictors for Model Development

The selection of predictor variables was based on five principles: (a) firmly established risk factors for recurrence, (b) easy to determine in clinical practice, (c) must be applicable in a broad range of patients. (d) the predictive value must be high in our own cohort, (e) maximum four predictors to avoid overfitting.

The following risk factors were considered because of previous publications: (1) Unprovoked DVT of the index event is considered to be the most important risk factor for recurrence. This was confirmed in many cohort studies in different settings and populations (14). In contrast to previous prediction models, we included this factor in the prediction model making it applicable to patients with provoked DVT as well. (2) Male sex is an established risk factor for VTE recurrence. A higher risk of recurrence in men was observed in a number of cohort studies and an individual-patient meta-analysis summarizing this evidence estimated a 2.2-fold higher risk in men compared to women (18). This variable was already implemented in two previous prediction models (Vienna prediction model; DASH score). (3) Elevated D-dimer measured 1 month after stopping anticoagulation is associated with an increased risk of recurrent VTE. A systematic review and meta-analysis summarizing the data estimated an 8.9% annual risk in patients with elevated D-dimer compared to 3.5% annual risk in patients without (19). We decided however not to include D-dimer in the prediction model in order to avoid the requirement of stopping the anticoagulation for 1 month. (4) Different cohort studies observed a higher risk of recurrence in patients with a high factor VIII compared to patients without (23, 24, 39). Presence of inflammation was defined as an active systematic inflammatory disorder such as inflammatory bowel disease or inflammatory rheumatologic disease (39).

### Model Building and Statistical Analysis

Numbers/ frequencies or median/ inter-quartile ranges was reported to describe patient characteristics. The subgroups “provoked by surgery,” “non-surgical transient risk factor,” “unprovoked VTE,” as well as “active cancer” are mutually exclusive groups. Incidence rates per 100 patient-years were reported.

A commonly used rule-of-thumb states that at least 10 events should be recorded for each predictor included in the analysis. We allowed a maximum of 4 predictors, corresponding to over 15 events per predictor variable. Incomplete predictor values were imputed using stochastic regression imputation to prevent a loss of statistical precision and to reduce the likelihood of selection bias. A cox proportional hazards model was used to determine associations between predictor variables and recurrent VTE. The analysis was adjusted for periods of anticoagulation by including this variable as a time-varying co-variable. We used stepwise forward selection to arrive at a model containing only predictors that contributed significantly to the model, using a relatively liberal *p*-value for selection of 0.10 to make sure potentially important predictors would not be omitted. Using the regression coefficients (i.e., the natural logarithm of the hazard ratios) we simplified the model into a score based on integers only by selecting the smallest whole numbers that would still preserve the relative differences in importance between predictors. To adjust for potential overfitting, standard bootstrapping techniques were used to internally validate the model. Using the 1,000 bootstrap samples, we corrected the optimism-corrected C-statistic, which is an estimation of the C-statistic when the model is applied to future patients. The C-statistic, or concordance statistic, is a measure of discrimination (i.e., the ability of the model to separate outcomes). Patients were ranked according to their risk score and we created three groups of roughly similar size. As long as a risk score that has no unit rather than a prediction model was build, we did not create calibration plots and did not calculate the agreement between predicted and observed outcomes. A Kaplan-Meier curve stratified by risk category was used instead to assess differences in time-to-event between risk groups. A sensitivity analysis was conducted after excluding cancer patients.

The R statistical package was used for analysis [R Development Core Team (2019). R: A language and environment for statistical computing. R Foundation for Statistical Computing, Vienna, Austria. ISBN 3-900051-07-0, URL http://www.R-project.org.].

## Results

### Patient Characteristics and VTE Recurrence

Four-hundred and seventy-nine patients were included in the study cohort and followed for a median of 3.12 years (IQR 0.78, 3.90) after stopping anticoagulation treatment. Patient characteristics are reported in Table 1. Five patients were lost to follow-up within the 2 years of CCP (1%; moved abroad), and 17 patients were lost during the extended follow-up (3.6%; Figure 1). Median age was 58.0 years (inter-quartile range, IQR 46.1, 71.1), and 242 were female (50.5%). All patients received vitamin K antagonists. Unprovoked DVT was present in 265 cases (55.3%). Sixty-four recurrent VTE were observed (25% of the patients), comprising 39 patients with DVT (60.9%), 20 patients with PE (31.3%), and 5 patients with other VTE (7.8%).

**Table 1:** Patient characteristics (*n* = 479)^*^.

**Characteristics**	**Frequency**	**Missing values**
	**Number (%)**	**Numbers**
Age (median, IQR)	58.0 (46.1, 71.1)	0
Females	242 (50.5)	0
**Risk factors**		
Provoked by surgery^°^	95 (19.9)	0
Non-surgical transient risk factor^°^	107 (22.3)	0
Unprovoked VTE^°^	265 (55.3)	0
Active cancer^°^	12 (2.5)	0
Pregnancy	6 (1.3)	0
Contraceptive use	50 (10.4)	6
Travel	34 (7.1)	0
Immobilization	40 (8.4)	6
Inflammation	64 (13.4)	7
Previous VTE	91 (19.0)	0
Cardiovascular disease	115 (24.0)	5
Heart failure	6 (1.3)	6
Known thrombophilia	19 (4.0)	7
Venous insufficiency	31 (6.5)	8
Varicosis	22 (4.6)	177
Residual thrombosis^†^	144 (30.1)	60
Smoking	107 (22.3)	29
Family history	140 (29.2)	14
Elevated D-dimer^&^	112 (23.4)	122
Elevated CRP^&^	109 (22.8)	174
Elevated FVIII^&^	65 (13.6)	157
Elevated Villalta score^$^	78 (16.3)	96

* *Observation period 2,231 patient-years; median 3.1 years; five patients were lost to follow-up because they moved abroad*;

° *mutually exclusive groups*;

† *determined 1 week before intended stop of anticoagulation treatment*;

& *1 month after stopping anticoagulation treatment*;

$ *at 6 months*.

### Predictors of Recurrent VTE

The full list of predictors analyzed are reported in a previous publication (15). Five predictors were statistically significant in univariate analysis: (1) unprovoked VTE, (2) male sex, (3) elevated D-dimer, (4) high factor VIII, and (5) presence of inflammation. The corresponding number of events, incidence rates per 100 patient-years, and unadjusted hazard ratios are reported in Table 2.

**Table 2:** Recurrence rates and hazard ratios according to predictor variables.

**Predictor variable**	**Events (numbers)**	**Incidence rate per 100 patient-years (95%CI)**	Hazard ratio^*^ (unadjusted; 95%CI)	**Points attributed in the prediction model^‡^**
All patients	64	2.9 (2.2, 3.7)	N/A	
Unprovoked DVT	47	4.0 (3.0, 5.3)	3.1 (1.3, 7.4)	2
Male sex	41	3.9 (2.9, 5.3)	2.0 (1.2, 3.3)	1
Elevated D-dimer+	26	5.1 (3.4, 7.4)	2.5 (1.5, 4.3)	
High factor VIII+	17	5.2 (3.2, 8.3)	2.3 (1.3, 4.2)	2
Presence of inflammation	15	4.7 (2.9, 7.9)	1.9 (1.1, 3.4)	

* *Cox proportional hazards model adjusted for periods of anticoagulation by including this variable as a time-varying co-variable; + assessed 1 month after stop anticoagulation treatment*;

‡ *patients will be categorized into three groups: low risk (score 0), medium risk (scores 1, 2, or 3) and high risk (scores 4 and 5)*.

### Development and Performance of the Prediction Model

**Figure 2:**
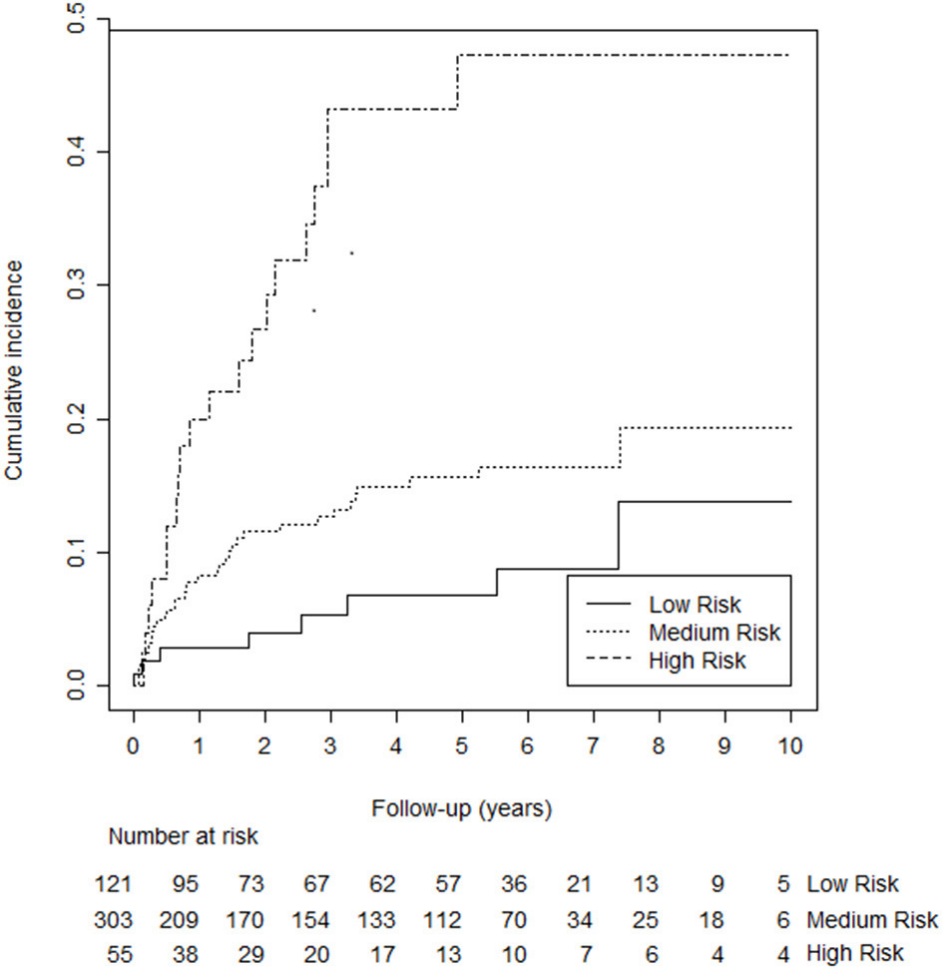
Kaplan-Meier curves illustrating cumulative recurrence according to risk categories of the Continu-8 score. The 5-years probability was 7.7% in the low-risk group (95% CI: 2.9–12.2%), 12.1% in the medium-risk group (95% CI: 4.2–19.3%), and 29.1% in the high-risk group (95% CI: 20.9–36.5%).

Using the predictors mentioned above, we added “unprovoked VTE,” “male sex,” “high factor VIII,” and “presence of inflammation” in a stepwise manner to the multivariable Cox proportional hazards model. We skipped D-dimer for practicability reasons (discussed above). The variable “presence of inflammation” and was omitted from the final model beause it did not improve the discrimitative ability. The adjusted hazard ratios (HR's) for unprovoked VTE, male sex, and high factor VIII were 2.19 [95% confidence interval (CI): 1.22–3.91, *p* = 0.008], 1.62 (95% CI: 0.95–2.77, *p* = 0.077), and 2.07 (95% CI: 1.23–3.47, *p* = 0.006), respectively. A sensitivity analysis after excluding cancer patients (*n* = 12) did not change these HR. After converting to integers, the score ranged from 0 to 5 and was categorized into three groups [i.e., low risk (score 0), medium risk (scores 1, 2, or 3) and high risk (scores 4 and 5)]. The 5-years probability of recurrence for the low, medium, and high-risk groups was 7.7% (95% CI: 2.9–12.2%), 12.1% (95% CI: 4.2–19.3%), and 29.1% (95% CI: 20.9–36.5%). Figure 2 illustrates the cumulative recurrence according to risk categories of the Continu-8 score. The optimism-corrected C-statistic of the Continu-8 score was 0.68 (95% CI 0.61, 0.75).

## Discussion

Using data from a prospective cohort study following patients with a proximal DVT, we developed and internally validated a potential new prediction model for recurrent VTE that can be used in patients with provoked and unprovoked proximal DVT without stopping anticoagulant treatment. The performance of the prediction model was adequate.

Our findings are essentially in-line with previous studies. The four most important predictors were already confirmed in other observational studies: (a) unprovoked DVT (2, 14, 17, 40, 41), (b) male sex (18), (c) elevated D-dimer (19), and (d) high factor VIII (22–24, 42, 43). In addition, inflammatory conditions were associated with recurrent VTE (44); this effect was however not significant anymore in multivariate analysis. In contrast to older studies, the risk of recurrence was very low in patients with pregnancy-related DVT or contraceptive use (45). In fact, the number of recurrent events is very low, leading to wide confidence intervals, and impeding the implementation in the prediction model. Our interpretation is that the awareness on the pregnancy and estrogen-related risk is much higher nowadays, leading to strict avoidance or medical prophylaxis in such patients.

Previous prediction models were developed in patients with unprovoked VTE only (25, 27, 29, 33). In contrast, we incorporated this variable in order to apply the model to all patients with proximal DVT, extending the prediction model to a more broader range of patients. Male sex was already included in the Vienna prediction model as well as the DASH score (29, 33). In contrast to HERDOO2 (27), Vienna prediction model (29, 30), and DASH (33), we did not include D-dimer in order to facilitate risk assessment without stopping anticoagulation treatment. High factor VIII was added to the DASH score in a sub-analysis of the MEGA follow-up study investigating the predictive value of factor VIII for recurrent VTE, what improved the c-statistics of the DASH score (22). We did also not include age (27, 33), body mass index (27), presence of post-thrombotic syndrome (27), and site of index event (29) in the model.

The strength of our investigation is that a relatively high number of events were available per predictor (64 events for 4 predictors studies), resulting in a considerable precision of the estimates. In addition, it was conducted in a reasonable number of patients, conducting a long-term follow-up, combined with a low number of patients lost. Of course, we are faced with limitations as well. First, only patients with (proximal) DVT were studied. At the present moment, the results of our study cannot be applied to patients with PE. We believe however that future external validations will confirm our results because previous prediction models using similar sets of predictors were generated in populations including PE (22, 27, 29, 30, 33). Secondly, a very low number of patients with cancer were included, preventing the application of the prediction model to this special group of patients. Thirdly, factor VIII was measured after stopping anticoagulation. Even though the impact of anticoagulation treatment on factor VIII measurements is assumed to be low, we cannot fully exclude that the results would be different while continuing anticoagulation treatment. Fourthly, due to the specific characteristics of the study population (proximal DVT as index event only, few cancer patients) and the predictor variables implemented in the model (unprovoked DVT, sex, increased coagulation activity), we were not able to conduct sensitivity analyses in meaningful subgroups of patients to assess the internal validity. However, our results are essentially in-line with previous studies suggesting external validity. Fifthly, the exact number of patients with distal DVT as the recurrent event type was not recorded. However, there were few patients only and we do not believe that this might have introduced any bias. Sixthly, even though bootstrapping techniques were used to adjust for potential overfitting, we cannot fully exclude that such effects might have affected the results. Seventhly, we did not discuss a risk-benefit trade-off to decide on the cut-off to be considered for prolonged anticoagulation. Given the apparent limitations of the study, this was beyond the focus of the current manuscript. Eighthly, we did not include age-adjusted D-Dimer cut-offs in the prediction model because it was beyond this manuscript's focus. Ninthly, our data were obtained with vitamin K-antagonists used in most patients and we cannot fully exclude that this might have affected the results.

What do the results of the study mean for scientific inquiry and clinical practice? First, our study confirms that prediction models or clinical prediction rules, respectively, can be applied to patients with DVT. Even though a c-value of 0.68 is not a high number, it is similar to previous prediction models (27, 29, 30, 33). Thus, the predictive ability of our score appears to be adequate and supports further investigation. Besides, it was possible to apply the prediction rule to all patients with proximal DVT, not only unprovoked DVT. Secondly, the set of predictors resembles previous prediction rules, verifying these results as well (27, 29, 30, 33). Third, the set of predictors incorporated in prediction models for recurrent VTE can be simplified to few variables that can be easily scored in clinical practice. Fourth, factor VIII measurements might replace D-dimer in order to prevent stopping anticoagulation. However, these results must be verified in patients with different settings and populations. In particular, future studies might confirm these results in patients without stopping anticoagulation while measuring factor VIII.

With the present investigation, we were able to develop and validate a new prediction model to be used in patients with both, provoked and unprovoked DVT, potentially without stopping anticoagulation treatment. Awaiting external validation in patients with PE and other populations and settings without stopping anticoagulation treatment, the prediction model has the potential to improve care in patients with VTE.

## Data Availability Statement

The raw data supporting the conclusions of this article will be made available by the authors, without undue reservation.

## Ethics Statement

The studies involving human participants were reviewed and approved by Medisch-ethische toetsingscommissie azM/UM (METC), number 15-4-256. Written informed consent for participation was not required for this study in accordance with the national legislation and the institutional requirements.

## Author Contributions

MN and AT developed the study design, collected the data, and wrote the manuscript. MN and SV developed the analysis plan. SV conducted the statistical analysis. AT developed and implemented the clinical care pathway and acted as principal investigator. MN, AT, SV, MP, and HT reviewed the study design and statistical analysis and wrote the manuscript. All authors contributed to the article and approved the submitted version.

## Conflict of Interest

MN reports receiving grants from the Swiss National Science Foundation (SNSF), during the conduct of the study; and research grants from Bayer, Stago, Roche diagnostics, outside of the submitted work. The remaining authors declare that the research was conducted in the absence of any commercial or financial relationships that could be construed as a potential conflict of interest.
